# Psoriasis and Psoriatic Arthritis in Pregnancy: Current Evidence and Clinical Considerations

**DOI:** 10.7759/cureus.116859

**Published:** 2026-09-24

**Authors:** Anna Paczkowska, Julia Danieluk, Michal Glinski, Natalia Adamaszek, Kinga Grabowska, Aleksander Gawda, Agata Nawrocka

**Affiliations:** 1 Dermatology, Pomeranian Medical University, Szczecin, POL; 2 Internal Medicine, Uniwersytecki Szpital Kliniczny w Poznaniu, Poznań, POL; 3 Medicine, Uniwersytecki Szpital Kliniczny w Poznaniu, Poznań, POL; 4 Pediatrics, Uniwersytecki Szpital Kliniczny w Poznaniu, Poznań, POL; 5 Medicine, Clinical Hospital of Poznań, Poznań, POL; 6 Medicine, Dr. Antoni Jurasz University Hospital No. 1 in Bydgoszcz, Bydgoszcz, POL

**Keywords:** fertility, lactation, preconception counselling, pregnancy, psoriasis, psoriatic arthritis, reproductive health

## Abstract

Psoriasis and psoriatic arthritis (PsA) are chronic, immune-mediated inflammatory diseases that commonly affect individuals of reproductive age, presenting unique challenges relating to fertility, pregnancy, lactation, and long-term reproductive health. This narrative review summarizes the current evidence on the relationship between psoriatic diseases and pregnancy, focusing particularly on disease activity, fertility, obstetric outcomes, hereditary risk, the psychosocial impact, and treatment options throughout the reproductive journey.

The available evidence indicates that psoriasis and PsA can impact reproductive health and fertility, as well as pregnancy outcomes, particularly in individuals with active or severe disease. Chronic systemic inflammation and associated comorbidities likely play an important role in these associations. While pregnancy-related immunological adaptations may improve disease activity in some women, flare-ups remain common during the postpartum period.

The management of psoriatic disease during pregnancy has undergone a substantial paradigm shift. Contemporary evidence increasingly supports maintaining adequate disease control throughout the reproductive period with therapies with an established safety profile.

This review highlights the evolving understanding of psoriasis and PsA as systemic diseases that extend beyond dermatological and rheumatological manifestations and may influence reproductive health at multiple levels. Future research should focus on the long-term safety of newer targeted therapies, the impact of paternal exposure, patient-reported reproductive outcomes, and developing evidence-based, multidisciplinary care pathways for women with psoriatic disease during pregnancy and the postpartum period.

## Introduction and background

Psoriasis is a chronic, relapsing, immune-mediated inflammatory disease in which abnormalities in T-cell responses, chronic inflammation, and excessive keratinocyte proliferation play a key role. The disease affects approximately 1-3% of the population, although its prevalence in Europe is estimated at around 1.8-2.0% [1,2]. Psoriasis can occur at any age, but it often begins in young adulthood: in men, especially between the ages of 15 and 25, and in women, with a peak incidence between the ages of 16 and 22 [2,3].

The significance of psoriasis in women of childbearing age extends beyond skin lesions. The disease, particularly in moderate and severe forms, can significantly impact quality of life, family planning decisions, and treatment choices [4]. Skin symptoms, such as erythematous-exfoliative plaques, itching, pain, and visible lesions, are associated with psychological and social distress. Furthermore, psoriasis is increasingly understood as a systemic disease rather than solely a dermatological one. Patients are at increased risk of comorbidities, including metabolic syndrome, diabetes, hypertension, cardiovascular disease, depression, and other autoimmune diseases [1,5,6].

A distinct disease phenotype is psoriatic arthritis (PsA). PsA is a chronic, seronegative inflammatory spondyloarthropathy that develops in approximately 20-30% of patients with psoriasis [7,8] and typically develops several years after the onset of skin lesions [3].

However, the course of psoriasis during pregnancy is variable and difficult to predict. In some patients, skin lesions improve; in others, the disease remains stable; while in the remainder, it may worsen. Systemic inflammatory diseases are particularly important in pregnancy. Chronic activation of the IL-23/Th17/IL-17 axis, characteristic of psoriasis, may contribute not only to persistent skin lesions but also to chronic inflammation throughout the body [7,9]. Elevated levels of pro-inflammatory cytokines may affect placental function, angiogenesis, and immunological balance at the maternal-fetal interface. For this reason, active inflammatory disease may be associated with an increased risk of obstetric complications, such as preterm birth, low birth weight, or preeclampsia [10].

The management of psoriasis and PsA in women planning pregnancy also remains a significant clinical challenge. Many patients of childbearing age require systemic treatment, and therapeutic decisions must balance both the risks associated with active disease and the potential risks of the therapy to the fetus. Discontinuation of systemic treatment before pregnancy or during the first trimester may lead to an exacerbation of disease activity, especially in the case of PsA [4]. At the same time, certain medications, such as methotrexate, acitretin, or leflunomide, are contraindicated during the period of pregnancy planning and require discontinuation well in advance [11].

Despite advances in the treatment of inflammatory skin and joint diseases, there is still a lack of clear, high-quality evidence-based guidelines for the management of pregnant women with psoriasis and PsA. This is mainly because few prospective studies involve pregnant women. In practice, physicians often base treatment decisions on clinical experience, product summaries, registry data, case reports, and observational studies [4]. This underscores the need for individualized treatment and close collaboration between a dermatologist, a rheumatologist, and an obstetrician-gynecologist.

This paper provides a concise overview of the current state of knowledge on the association between psoriasis and PsA and reproductive health, with a particular focus on fertility, disease course, pregnancy outcomes, postpartum health, and lactation. It is imperative to emphasize the importance of adhering to the principles of safe, individualized therapeutic management throughout the reproductive period. Multidisciplinary care is also paramount.

The present narrative review was conducted using literature identified through searches of PubMed/MEDLINE and Google Scholar. The literature search primarily covered English-language publications from January 2000 to June 2026. The final literature search was conducted in June 2026. Earlier studies were included in the present review if they were considered to be clinically relevant or important for the interpretation of current evidence and recommendations.

The search strategy combined terms related to psoriasis, PsA, reproductive health, fertility, conception, preconception care, pregnancy, pregnancy outcomes, obstetric outcomes, postpartum disease activity, lactation, breastfeeding, systemic therapy, and biologic therapy. The principal search terms included "psoriasis", "psoriatic arthritis", "pregnancy", "fertility", "conception", "preconception", "pregnancy outcomes", "obstetric outcomes", "postpartum", "lactation", "breastfeeding", "systemic therapy", "biologic therapy", and "reproductive health". The combination of these terms was achieved by employing the Boolean operators AND and OR. A further series of publications was identified through a manual screening of the reference lists of relevant articles, reviews, and international clinical guidelines.

The following publications were deemed eligible for inclusion: observational studies, cohort studies, case-control studies, clinical studies, registry-based studies, systematic reviews, meta-analyses, narrative reviews, consensus statements, and clinical practice guidelines. We included studies addressing psoriasis and/or PsA in relation to fertility, preconception care, pregnancy, postpartum disease activity, obstetric outcomes, lactation, breastfeeding, or treatment safety. We excluded publications not relevant to reproductive health, pregnancy, lactation, or managing psoriasis or PsA in women of reproductive age.

The selection process began with title and abstract screening, followed by full-text review of potentially eligible articles. When multiple publications addressed a common clinical query, we prioritized prevailing international guidelines, systematic reviews and meta-analyses, large cohort and registry studies, and studies with pregnancy-specific data. As this was a narrative review, we did not perform a formal risk-of-bias assessment or quantitative evidence synthesis.

When disease-specific data were available, we evaluated evidence on psoriasis and PsA separately. This was particularly true for disease activity during pregnancy and the postpartum period, pregnancy and obstetric outcomes, and treatment. Joint consideration of evidence was contingent on studies or guidelines addressing psoriatic disease or on the absence of available disease-specific data.

The selected evidence was narratively synthesized by reproductive timeline, with particular emphasis on preconception, pregnancy, delivery and neonatal considerations, and the postpartum and breastfeeding periods. The synthesis focused on fertility, disease activity, pregnancy and obstetric outcomes, treatment safety, placental transfer of biologic therapies, infant vaccination, and contemporary management strategies.

The extant evidence demonstrates that pregnancy in women with psoriasis and PsA is influenced by the complex interaction between disease-related inflammation, comorbidities, and therapeutic interventions. Whilst pregnancy is commonly associated with positive outcomes, patients with active or severe disease appear to be at increased risk of maternal and obstetric complications. It is important to note that disease activity may either improve, remain stable, or worsen during pregnancy; however, exacerbations are frequently reported postpartum. A growing body of evidence supports selected biologic therapies and underscores the importance of personalized treatment strategies throughout the reproductive period.

## Review

Epidemiology of psoriasis and PsA in women of childbearing age

Psoriasis is one of the most common chronic inflammatory skin diseases affecting women of childbearing age, who account for approximately 15-20% of the total population with psoriasis [12]. 82% of women experience at least one pregnancy in their lifetime. It has been estimated that 65,000-107,000 births per year involve women with psoriasis, of which approximately 9,000-15,000 are to patients with moderate or severe disease [13].

These data underscore the importance of proper treatment planning during the preconception period and throughout pregnancy. Additionally, approximately 40-45% of pregnancies worldwide are unintended, which is a significant argument for considering reproductive safety when evaluating women for systemic and biologic therapies [14].

Pathogenesis of psoriasis and PsA and immunological changes during pregnancy

Psoriasis is a chronic, systemic inflammatory disease with a complex etiology involving genetic, immunological, and environmental factors [7,15,16]. Genetic susceptibility contributes, particularly the HLA-C06:02 allele in the PSORS1 region, which has been linked to early-onset disease and type I psoriasis [16]. Conversely, the HLA-B27, HLA-B38, HLA-B39, and HLA-B08 alleles have been linked to an elevated risk of joint involvement and axial symptoms [8].

Beyond genetic predisposition, environmental factors influence psoriasis development, including streptococcal infections, psychological stress, smoking, alcohol consumption, obesity, and alterations in the gut microbiota [7,17]. Obesity is a particularly salient factor, given the propensity of adipose tissue to produce pro-inflammatory cytokines, including TNF-α and IL-6. Moreover, an elevated BMI has been demonstrated to be associated with an increased risk of psoriasis and greater disease activity [7,17]. Chronic inflammation may promote metabolic disturbances that can affect pregnancy outcomes and increase the risk of obstetric complications [7,8]. Diet and gut dysbiosis may also influence disease activity, while certain medications, including β-blockers, lithium salts, antimalarial drugs, and interferons, may induce or exacerbate psoriasis [8,17].

Skin trauma (the Koebner phenomenon) and mechanical microtrauma to tendon and ligament insertions have been observed to trigger localized inflammation in PsA ("deep Koebner phenomenon"), which may partially explain the observed association between mechanical overload and an increased risk of developing the disease [8].

A key aspect of psoriasis and PsA pathogenesis is the activation of innate and adaptive immune responses, with the IL-23/IL-17 axis playing a particularly pivotal role. IL-23, predominantly produced by dendritic cells and macrophages, is critical for activating and maintaining Th17 lymphocytes. These cells, in turn, produce cytokines such as IL-17 and IL-22 [1,8]. In conjunction with TNF-α, these cytokines perpetuate chronic inflammation and keratinocyte proliferation; in PsA, they contribute to synovial and tendinous inflammation, as well as structural changes in the joints and bones [8,15]. Th17 and Treg lymphocytes are also implicated in pathogenesis, and an imbalance in the Th17/Treg ratio has been shown to further contribute to chronic inflammation [1].

Pregnancy represents a unique immunological state that requires a balance between tolerance towards the semi-allogeneic fetus and an effective anti-infective response [18,19]. Unlike global immunosuppression, pregnancy features dynamic modulation of innate and adaptive immunity. This includes increased regulatory T-cell activity, a more tolerogenic cytokine profile involving IL-10 and TGF-β, and relative suppression of Th1 and, to some extent, Th17 responses [18,19]. These changes vary across pregnancy.

During the first trimester of gestation, a regulated pro-inflammatory response is imperative for embryo implantation and placental development [20]. A relative predominance of the Th1 response, involving IFN-γ and TNF-α, supports trophoblast invasion and spiral artery remodeling. In contrast, an expanding population of CD4+CD25+FoxP3+ regulatory T-cells regulates Th17 activity, maintaining the balance between inflammation and fetal tolerance [20].

During the second and third trimesters, increasing levels of estrogens, progesterone, and human chorionic gonadotropin (hCG) promote a more tolerogenic immune environment [20,21]. Estrogens and progesterone promote Th2 and Treg responses while suppressing Th1 and Th17 activity. For instance, estrogens inhibit Th17 differentiation, while progesterone reduces IL-17 production and promotes Treg development [20]. Because psoriasis and PsA are driven largely by the IL-23/Th17 axis and IL-17 production, attenuating the Th17 response during pregnancy may contribute to clinical improvement [20].

Increasing levels of endogenous glucocorticoids may further contribute to psoriasis remission, while prolactin, human placental lactogen, and hCG also exert immunomodulatory effects [21]. However, it is acknowledged that some patients experience disease exacerbation during pregnancy, the mechanisms of which remain incompletely understood [21].

Following delivery, the rapid decline in estrogen and progesterone levels has been demonstrated to promote reactivation of Th1 and Th17 responses, which may contribute to exacerbations of psoriasis and PsA postpartum [20]. A considerable proportion of women experience recurrence or exacerbation of skin lesions and increased joint activity postpartum [22], emphasizing the necessity for adequate postpartum monitoring and treatment planning, particularly during breastfeeding.

Course of psoriasis and PsA during pregnancy and after childbirth

The clinical course of psoriasis and PsA during pregnancy is variable and difficult to predict. Skin lesions improve in approximately 55-60% of pregnant women, stabilize in approximately 20%, and worsen in 20-26% [11,20]. Reduced disease activity may be associated with the relative suppression of Th1 and Th17 responses during pregnancy [20]. However, exacerbations are common postpartum, with new or worsening skin lesions emerging within six to eight weeks after delivery in 62-87% of patients [20], while approximately 65% of women report exacerbation of plaque psoriasis or PsA [11]. Furthermore, the postpartum period has been observed to coincide with the onset of PsA, with 30-40% of women attributing the emergence of joint symptoms to this phase [13].

The course of PsA may be particularly unpredictable, with joint inflammation persisting or worsening during pregnancy, especially following treatment discontinuation before conception or in early pregnancy [11]. In a prospective study by Polachek et al., disease activity remained stable or increased in a substantial proportion of patients, and approximately 40% experienced moderate or high disease activity postpartum [22]. Exacerbation of joint symptoms postpartum has been reported in 65-75% of women, possibly due to hormonal changes and reactivation of Th1/Th17 responses [23]. The registry study by Broms et al. further indicated an increased need for treatment intensification after delivery, with the highest risk of disease flares occurring during the first six months postpartum [24].

The effective control of PsA prior to pregnancy has been demonstrated to reduce the risk of flares during pregnancy and the postpartum period. Conversely, abrupt treatment discontinuation has been shown to increase the risk of arthritis recurrence and deterioration in physical functioning [22,25]. These findings underscore the importance of personalized treatment regimens and close collaboration among dermatologists, rheumatologists, and obstetrician-gynecologists.

Assessment of disease activity

Women with psoriasis and PsA should undergo rigorous clinical monitoring before and during pregnancy [26]. Effective disease control before conception may reduce the risk of flares during pregnancy and the postpartum period and potentially mitigate adverse obstetric outcomes. Psoriatic diseases have been associated with an increased risk of cesarean delivery, preterm birth, preeclampsia/eclampsia, gestational diabetes, and gestational hypertension [26].

In cutaneous psoriasis, clinicians assess disease severity using the Psoriasis Area and Severity Index (PASI), body surface area (BSA), and the Dermatology Life Quality Index (DLQI). PASI evaluates lesion severity and extent (range 0-72), BSA reflects the percentage of affected skin, and DLQI assesses the impact of disease on quality of life [27]. According to the Polish Dermatological Society, psoriasis is categorized as mild when PASI, BSA, and DLQI are all <10. In contrast, a value ≥10 in at least one index indicates moderate-to-severe disease and may necessitate phototherapy or systemic treatment [28]. Treatment efficacy can be evaluated by measuring PASI reduction, with a PASI-90 response considered excellent. If improvement is insufficient after three to four months, treatment modification is advised [28]. During pregnancy planning, these measures support disease monitoring and selection of appropriate therapy, although evidence directly linking their values to specific obstetric outcomes remains limited [27].

Assessing PsA is challenging because it is multidomain, encompassing peripheral and axial arthritis, enthesitis, dactylitis, and skin and nail involvement. The primary function of the DAPSA is to assess peripheral joint disease; however, it is not well equipped to capture axial disease, enthesitis, dactylitis, or skin involvement [29]. PASDAS provides a more extensive multidomain evaluation; however, it is more intricate and less practical for routine clinical application. Minimal disease activity and very low disease activity incorporate several PsA domains and are useful treatment targets, although they are categorical states rather than continuous measures of disease activity.

DAS28-CRP, despite its accessibility and use in select PsA studies, was originally developed to evaluate rheumatoid arthritis. It is limited in its capacity to assess a comprehensive range of joints, resulting in an inadequate evaluation of pivotal PsA domains, including distal interphalangeal joints, enthesitis, dactylitis, axial disease, and skin involvement [29]. Similarly, BASDAI was developed for ankylosing spondylitis and may assess axial symptoms; however, it does not comprehensively capture PsA activity. Consequently, a single index cannot fully capture the heterogeneous manifestations of PsA, and disease assessment should incorporate relevant clinical domains. Evidence on the use and validation of these instruments during pregnancy remains limited [29].

Impact of disease on women's fertility

Given that psoriasis is a chronic systemic disease associated with persistent inflammation, metabolic disorders, and a considerable psychosocial burden, it may affect fertility [20,30,31].

One key mechanism that may affect fertility in women with psoriasis and PsA is disruption of the hypothalamic-pituitary-ovarian (HPO) axis. Chronic inflammation increases pro-inflammatory cytokines such as TNF-α and IL-6, which can affect gonadotropin-releasing hormone secretion, disrupt LH and FSH secretion, and impair ovarian steroidogenesis [20,31,32]. Pro-inflammatory cytokines may also negatively affect folliculogenesis, oocyte quality, and endometrial receptivity [20,31]. These mechanisms are particularly relevant in moderate and severe psoriasis and in patients with PsA, in whom chronic inflammation is more intense and systemic.

Psychosocial factors also contribute substantially to fertility disorders. As a visible, chronic, and frequently socially stigmatized condition, psoriasis can adversely affect mental well-being, increasing the risk of depression, anxiety, chronic stress, and diminished self-esteem [20,30]. It is hypothesized that chronic stress and depression can further affect the functioning of the HPO axis by disrupting neuroendocrine balance and exacerbating inflammatory processes [20,31]. Psychological distress also affects interpersonal relationships, sexual functioning, and decisions regarding motherhood.

In a retrospective observational study by Burlando et al. involving 100 women with psoriasis, 73 women reported a history of pregnancy, accounting for 170 pregnancies in total. The mean number of children per woman was 1.2 ± 1, which was lower than the national average. Miscarriage occurred in 18.2% of pregnancies, with a higher proportion reported among women with concomitant PsA (27.4%) [30]. The authors suggested these findings may relate to the greater systemic inflammatory burden associated with joint involvement. At the same time, reproductive decisions may also be influenced by impaired quality of life and concerns regarding pregnancy and treatment safety [30]. A review by Eudy et al. summarized previous studies indicating that infertility was reported in approximately one-third of women with PsA, with polycystic ovary syndrome being one of the most frequently reported contributing factors [31]. Women with PsA also more frequently reported concerns regarding medication safety, their ability to care for a child, and the potential impact of the disease on pregnancy [31].

Furthermore, data from the BIOBADADERM registry demonstrated a more than 50% reduction in fertility rates among women with moderate-to-severe psoriasis compared with the general population [33]. However, not all studies have demonstrated a clear effect of psoriasis on reproductive outcomes. Lambe et al. found no significant difference in the total number of children between women with psoriasis and the control population. However, a higher proportion of women with psoriasis were childless [34]. Family-planning decisions may also be influenced by the need for systemic treatment and concerns regarding medication safety before conception and during pregnancy.

Paternal exposure and male fertility

In recent years, the impact of psoriasis and PsA on male reproductive health has received increasing attention. The chronic systemic inflammation associated with these diseases may reduce sperm quality by increasing oxidative stress, causing hormonal imbalances, and raising pro-inflammatory cytokine levels.

A study by Caldarola et al. found that men with psoriasis had a higher prevalence of accessory gland inflammation and impaired semen parameters than healthy controls. More recently, a Mendelian randomization analysis by Ma et al. suggested a causal link between psoriasis and lower sperm concentration, motility, and normal morphology. This supports the idea that chronic inflammation can negatively impact male reproductive function [35,36].

Concerns have also been raised regarding the effects of systemic therapies on male fertility and pregnancy outcomes. However, current evidence suggests that exposure to methotrexate in fathers is not associated with an increased risk of congenital malformations, miscarriage, or adverse birth outcomes. Consequently, contemporary rheumatology guidelines no longer recommend routinely discontinuing methotrexate in men planning fatherhood [37-39].

Studies evaluating TNF-α inhibitors and other biologic agents have not demonstrated deterioration in sperm concentration, motility, or morphology. Although no evidence suggests that biologic therapy directly enhances fertility, suppressing systemic inflammation may stabilize or improve selected semen parameters, particularly sperm motility, in men with active inflammatory disease [39,40].

Impact of psoriasis and PsA on pregnancy outcomes and obstetric results

In recent years, a growing body of research has indicated that psoriasis and PsA may affect the course of pregnancy and increase the risk of adverse obstetric outcomes. Chronic inflammation and elevated levels of pro-inflammatory cytokines, such as TNF-α and IL-6, are particularly important. These cytokines are also elevated in the umbilical cord blood of newborns born preterm or with low birth weight [20]. Since the intensity of the inflammatory response correlates with the severity of psoriasis, women with moderate-to-severe disease are at higher risk of developing pregnancy complications [20].

In a retrospective cohort study by Lima et al. involving 162 pregnancies in 122 women with psoriasis and 501 pregnancies in 290 women without psoriasis, a composite adverse pregnancy outcome comprising preterm birth (<37 weeks of gestation) and low birth weight (<2,500 g) occurred in 14.2% of pregnancies in women with psoriasis compared with 8.0% in controls (OR 1.89, 95% CI 1.06-3.39) [41-44]. The association remained significant after adjustment for relevant covariates (adjusted OR 1.95, 95% CI 1.10-3.46) [43,44]. At the same time, data regarding mild psoriasis are more reassuring. In a nationwide population-based study including 1,463 women with psoriasis and 11,704 controls, Yang et al. found that severe psoriasis was associated with an increased risk of low birth weight (adjusted OR 1.40, 95% CI 1.04-1.89). In contrast, mild psoriasis was not associated with a significant increase in low birth weight, preterm birth, cesarean delivery, small-for-gestational-age infants, or preeclampsia/eclampsia [41]. Notably, a large Danish prospective study involving over 100,000 pregnancies did not identify psoriasis as a risk factor for fetal loss after the first trimester [24].

Studies have also shown that women with PsA are more likely to develop other gestational complications, including gestational diabetes, pregnancy-induced hypertension, preeclampsia, and the need for elective or emergency cesarean section [20]. The risk of these complications was even higher in patients with severe psoriasis [20].

The higher burden of comorbid conditions observed in women with psoriasis should also be considered a contributing factor to adverse pregnancy and obstetric outcomes. Women with psoriasis are often older at the time of conception, have a higher body mass index, and more frequently suffer from diabetes, hypertension, depression, and metabolic syndrome compared with healthy pregnant women [20]. Additionally, unhealthy behaviors such as smoking and alcohol consumption are more prevalent in this population [20,45]. These metabolic, behavioral, and psychosocial factors are independently associated with an increased risk of obstetric complications, including preeclampsia, gestational diabetes, fetal macrosomia, and the need for cesarean delivery [20,41,42,44]. Furthermore, women diagnosed with psoriasis exhibited a lower propensity to utilize periconceptional vitamin or folic acid supplementation (53% vs. 68% of controls). This phenomenon may be partly attributed to a higher prevalence of unplanned pregnancies [45]. This is clinically significant because adequate periconceptional folic acid supplementation reduces the risk of neural tube defects [46].

A growing body of evidence suggests that active, uncontrolled disease may pose a greater risk of adverse pregnancy outcomes than appropriately monitored anti-inflammatory treatment [20]. Therefore, controlling peripheral and axial inflammation is particularly important in patients with PsA, as active systemic disease may increase the risk of exacerbations during pregnancy and the postpartum period [47].

Achieving remission or low disease activity prior to conception should therefore be one of the main goals of management in women with psoriasis and PsA planning a pregnancy. Properly controlled therapy, particularly biologic therapy with a documented safety profile, may be more beneficial than discontinuing treatment and losing control of the inflammatory process [20].

Heredity of psoriasis and PsA

Psoriasis and PsA are chronic inflammatory diseases with complex genetic and immunological underpinnings. Population-based studies demonstrate a strong familial aggregation of psoriasis. Individuals with an affected first-degree relative have an approximately 4-6-fold higher risk of developing psoriasis compared with the general population, whereas the risk remains moderately elevated among second-degree relatives [48]. Family studies have demonstrated that the risk of developing psoriasis increases substantially in individuals with affected parents, reaching approximately 40% when both parents are affected and 14% when only one parent has psoriasis, compared with a prevalence of approximately 2-3% in the general population [7,49]. In families of patients with PsA, psoriasis prevalence among first-degree relatives has been reported to reach 15-20%, while PsA itself occurs in approximately 7-10% of first-degree relatives [50]. Twin studies further support the importance of heredity, consistently showing substantially higher concordance rates among monozygotic twins than among dizygotic twins, with estimated heritability ranging from 66% to 90% [51]. Psoriasis is currently accepted as a polygenic, multifactorial disease that results from interactions between numerous genetic variants with small effects and environmental factors [52]. The strongest genetic risk signal is in the PSORS1 region within the major histocompatibility complex on chromosome 6p. The HLA-C*06:02 allele is particularly significant, as it is strongly associated with familial occurrence of the disease and early onset [53]. Modern genome-wide association studies (GWAS) and genetic meta-analyses have identified over 100 loci associated with psoriasis susceptibility [52]. Many of these loci involve genes regulating immune responses, including NF-κB signaling and antigen presentation pathways [50,52]. Immunogenetic and transcriptomic studies further support the role of genetically determined immune dysregulation in psoriasis development, linking susceptibility loci to the molecular mechanisms underlying the disease [52].

Interestingly, specific genetic variants are more strongly associated with particular clinical phenotypes, such as earlier disease onset or a more severe disease course [53]. The HLA-C*06:02 allele, located within the PSORS1 locus, is strongly associated with early-onset psoriasis (type I psoriasis), positive family history, and more extensive plaque psoriasis [52,53]. In contrast, patients negative for HLA-C*06:02 more frequently present with later-onset disease and a higher prevalence of metabolic comorbidities [52]. Polymorphisms in IL23R and IL12B are associated with chronic plaque psoriasis and increased susceptibility to PsA [50]. Nail psoriasis and axial involvement in PsA are linked more strongly to HLA-B27 and HLA-B38 alleles, whereas HLA-B08 has been associated with peripheral polyarticular forms of PsA [50]. Moreover, variants affecting NF-κB signaling and TNF-mediated pathways may contribute to more severe inflammatory phenotypes and differential therapeutic responses [50].

A significant genetic basis is also observed in PsA. Approximately 30% of patients with cutaneous psoriasis develop PsA [50]. This suggests a partially shared biologic and genetic predisposition between the two disease entities. The genetic risk for PsA partially overlaps with that for cutaneous psoriasis; however, specific markers associated with joint involvement have also been identified, including the HLA-B and IL23R genes [50]. GWAS analyses suggest that specific genetic markers may help identify patients with plaque psoriasis at higher risk of developing arthritis [50].

Genes increase the risk of developing the disease, but its manifestation requires interaction with additional environmental factors such as infections, smoking, obesity, psychological stress, and immune dysregulation [50,52,53]. For this reason, genetic counseling for patients should be based on a reliable presentation of the actual, yet non-deterministic, risk of inheriting the disease. Preconception counseling is an essential component of care for both women and men with psoriasis and PsA planning to have children [54]. The modern approach emphasizes interdisciplinary collaboration among a dermatologist, an obstetrician-gynecologist, and, in cases of coexisting PsA, a rheumatologist [54]. Patient education should include a discussion of actual family risk, the impact of lifestyle on disease progression, and the importance of controlling inflammation before planned pregnancy [54].

Psychosocial aspects

The coexistence of psoriatic disease and pregnancy represents a significant psychosocial burden. It has been demonstrated that gestation itself is associated with an elevated risk of developing mood and anxiety disorders. Moreover, the presence of chronic inflammatory disease has been shown to further compromise psychological well-being and quality of life [55].

Increased psoriasis severity has been demonstrated to be associated with a diminished quality of life and elevated psychological distress, with elevated PASI scores correlating with higher DLQI scores, depression, and anxiety [7,56]. In a study of 120 pregnant women with psoriasis, Dey et al. reported that 58.3% of patients had DLQI scores >10, with a mean score of 11.8 ± 5.6, while nearly 60% experienced moderate-to-severe psychological distress [55]. These factors particularly affect psychological well-being, self-esteem, social interactions, and intimate relationships [55].

Patients with psoriasis have an approximately 1.5- to 2-fold increased risk of developing depression and anxiety, particularly in cases of severe disease and concomitant PsA [56]. PsA is associated with an even greater psychosocial burden, with depression affecting approximately 30-40% and anxiety 20-30% of patients [57]. PsA is associated with a poorer physical and mental quality of life, greater work and social impairment, and difficulties related to pain, reduced mobility, body image, sexual well-being, and interpersonal relationships when compared with skin-limited psoriasis [57,58].

Visible skin lesions have been demonstrated to contribute to stigmatization, shame, reduced self-esteem, impaired body image, and social isolation [55,59]. Pregnant women with psoriasis have been reported to experience higher levels of psychological distress and poorer quality of life than healthy pregnant controls [55].

Concerns related to pregnancy include the exacerbation of pre-existing diseases, adverse obstetric outcomes, fetal health, and the safety of systemic and biologic therapies [55,60]. The lack of pregnancy-specific evidence for certain treatments may increase uncertainty among patients and healthcare professionals [60,61]. It is therefore imperative that appropriate preconception and pregnancy counseling be provided, including individualized assessment of treatment risks and benefits [61].

Social support, patient education, and multidisciplinary care have been shown to reduce psychological distress and enhance coping mechanisms and treatment adherence [55,60,61]. Consequently, the integration of routine screening for depression and anxiety, in conjunction with interdisciplinary collaboration among dermatologists, rheumatologists, obstetrician-gynecologists, and mental health professionals, is imperative [55].

Treatment of psoriasis and PsA during pregnancy

Topical Treatment

Topical treatment is first-line therapy for pregnant women with psoriasis, particularly for limited lesions and mild disease [13,62-64]. Preparations with minimal systemic absorption are preferred, as this helps reduce the potential risk of teratogenic effects [62,63]. The foundation of therapy includes emollients and moisturizers, which improve skin barrier function, reduce itching, and alleviate dryness while being well tolerated and posing no significant risk to the fetus [13,62,63].

The safety of topical glucocorticosteroids during pregnancy depends on using the lowest effective potency for the shortest necessary duration [13,62,63]. Mild- to moderate-potency corticosteroids are preferred, whereas potent and very potent preparations should be used with caution, particularly over large body areas or for prolonged periods [13,62,63]. Although earlier studies suggested a potential association between extensive use of potent or very potent topical corticosteroids and low birth weight, subsequent data have not demonstrated a substantial increase in adverse pregnancy outcomes [65].

Topical calcineurin inhibitors, including tacrolimus 0.03% or 0.1% ointment and pimecrolimus 1% cream, have not been approved for the treatment of psoriasis. However, they may be used off-label, particularly for facial, intertriginous, and genital psoriasis, where prolonged topical corticosteroid use carries a risk of skin atrophy [13]. Systemic absorption after topical application is generally minimal. Despite the paucity of pregnancy-specific data, topical calcineurin inhibitors may be considered when clinically indicated, particularly for intermittent treatment of limited skin areas [13,62,63].

Some topical preparations are contraindicated or should be used with great caution. Tazarotene, a topical retinoid, is contraindicated during pregnancy due to its potential teratogenic effects [13]. Similarly, the use of coal tar, salicylic acid, and vitamin D analogs is limited, particularly at high doses and on extensive skin areas [13].

Phototherapy

UVB phototherapy, particularly NB-UVB (311 nm), is considered first-line therapy for pregnant women requiring systemic treatment or with extensive skin lesions [13,62,63]. This therapy is not associated with an increased risk of congenital disabilities or preterm births and is considered the safest method for treating moderate-to-severe psoriasis during pregnancy [13]. NB-UVB can be used alone or in combination with topical treatment [62,63]. Compared to broadband UVB, it demonstrates greater therapeutic efficacy and a better safety profile [13]. However, PUVA phototherapy is not recommended during pregnancy because psoralens may be mutagenic and teratogenic [13,62,63].

A significant concern with UVB phototherapy is that UV radiation may reduce folic acid levels [13,62,63]. Folate deficiency may increase the risk of neural tube defects, particularly in early pregnancy. For this reason, folic acid supplementation of 4-5 mg/day is recommended during UVB therapy, along with monitoring of folate levels [62,63]. Additionally, overheating of the body should be avoided in the first weeks of pregnancy, as hyperthermia may further increase the risk of fetal developmental abnormalities [13].

Conventional Systemic Therapy

Conventional systemic therapy is primarily used in patients with severe psoriasis or active PsA when topical treatment and phototherapy are insufficient [62,63]. During pregnancy, however, treatment options are significantly limited due to the potential teratogenic effects of some medications. Table 1 summarizes current recommendations for systemic therapies during pregnancy.

**Table 1:** Safety of systemic therapies for psoriasis and PsA during pregnancy and lactation Recommendations should be individualized based on disease activity, prior treatment response, maternal comorbidities, and current product information. TNFi: tumor necrosis factor inhibitors, IL: interleukin, bDMARD: biologic disease-modifying antirheumatic drug, PsA: psoriatic arthritis

Medication	Preconception	Pregnancy	Breastfeeding	Placental transfer/fetal exposure	Infant vaccination considerations	References
Methotrexate	Discontinue before conception. Effective contraception is required during treatment and for at least six months after discontinuation, in accordance with current product information.	Contraindicated.	Contraindicated/avoid breastfeeding.	Pregnancy exposure should be avoided because of embryotoxicity and teratogenicity.	Routine schedule; in utero exposure should not occur.	[11,25,37]
Acitretin	Effective contraception during treatment and for three years after discontinuation.	Contraindicated.	Avoid/contraindicated.	Not applicable to biologic transfer; highly teratogenic.	Routine schedule; in utero exposure should not occur.	[1,3,12]
Sulfasalazine	Compatible; folic acid supplementation recommended.	Compatible.	Compatible.	Crosses placenta; clinically relevant neonatal immunosuppression is not expected.	Routine schedule.	[25,37,38]
Cyclosporine	May be continued when clinically indicated.	May be used when clinically indicated; monitor blood pressure and renal function.	Compatible with monitoring.	Crosses placenta.	Routine schedule.	[11,13,25,37,38]
Leflunomide	Discontinue before conception; accelerated elimination procedure when indicated.	Avoid/not recommended.	Not recommended.	Placental exposure is possible; pregnancy exposure should be avoided.	Routine schedule; in utero exposure should not occur.	[11,13,25,37]
Apremilast	Discontinue when pregnancy is planned.	Not recommended; insufficient human data.	Not recommended; insufficient data.	Limited/unknown data.	Routine schedule; no biologic-related restriction established.	[1,11,37]
Systemic glucocorticoids	No specific washout; not routinely recommended for plaque psoriasis.	Consider short-term use when clinically necessary, particularly in PsA; use the lowest effective dose for the shortest duration.	Generally compatible.	Crosses the placenta; fetal exposure varies by agent.	Routine schedule.	[62]
Adalimumab	No washout required if treatment is needed.	Compatible when clinically indicated.	Compatible.	Substantial active transfer, especially in the second and third trimesters.	After late-pregnancy exposure, follow current agent- and exposure-specific guidance for live vaccines.	[1,11,25,37,38]
Infliximab	No washout required if treatment is needed.	Compatible when clinically indicated.	Compatible.	Substantial active transfer, especially in the second and third trimesters.	After late-pregnancy exposure, follow current agent- and exposure-specific guidance for live vaccines.	[1,11,25,37,38]
Etanercept	No washout required if treatment is needed.	Compatible when clinically indicated.	Compatible.	Lower placental transfer than monoclonal IgG1 TNFi.	Live-vaccine timing should reflect gestational timing of exposure and current guidance.	[1,11,25,37,38]
Certolizumab pegol	No washout required if treatment is needed.	Compatible when clinically indicated; useful when continuous treatment throughout pregnancy is required.	Compatible.	Minimal placental transfer because it lacks an Fc region.	Routine vaccination is generally possible; follow current local/agent-specific guidance.	[1,11,25,37,38]
Ustekinumab	Individualized assessment.	Consider if clinically required; pregnancy evidence is less extensive than for TNFi.	Compatible according to current guidance; direct lactation data remain limited.	Active IgG transfer is expected and increases later in pregnancy.	Individualize live-vaccine timing after late-pregnancy exposure.	[11,37,38]
Secukinumab	Individualized assessment.	Consider if clinically required; pregnancy data remain limited.	Compatible according to current guidance; direct lactation data remain limited.	Active IgG transfer is expected, particularly later in pregnancy.	Individualize live-vaccine timing after late-pregnancy exposure.	[1,11,37,38]
Ixekizumab	Individualized assessment.	Consider if clinically required; pregnancy data remain limited.	Compatible according to current guidance; direct lactation data remain limited.	Active IgG transfer is expected, particularly later in pregnancy.	Individualize live-vaccine timing after late-pregnancy exposure.	[1,11,37,38]
Guselkumab	Individualized assessment.	Use only if clinically required after individualized risk-benefit assessment; data remain limited.	Compatible according to current guidance; direct lactation data remain limited.	Active IgG transfer is expected, particularly later in pregnancy.	Individualize live-vaccine timing after late-pregnancy exposure.	[11,38]
Tildrakizumab	Individualized assessment.	Use only if clinically required after individualized risk-benefit assessment; data remain limited.	Compatible according to current guidance; direct lactation data remain limited.	Active IgG transfer is expected, particularly later in pregnancy.	Individualize live-vaccine timing after late-pregnancy exposure.	[11,38]
Risankizumab	Individualized assessment.	Use only if clinically required after individualized risk-benefit assessment; data remain limited.	Compatible according to current guidance; direct lactation data remain limited.	Active IgG transfer is expected, particularly later in pregnancy.	Individualize live-vaccine timing after late-pregnancy exposure.	[11,38]

Methotrexate is contraindicated during pregnancy because of its teratogenic and embryotoxic effects related to folate antagonism and impaired DNA synthesis [13,62,63]. Fetal exposure may result in methotrexate embryopathy, including craniofacial, limb, and cardiac abnormalities [13]. Discontinue methotrexate before planned conception, per current guidelines and product-specific recommendations, and do not use it during breastfeeding [13,62,63].

Acitretin is highly teratogenic and contraindicated during pregnancy. Fetal exposure may cause retinoid embryopathy, including central nervous system, craniofacial, cardiac, and thymic abnormalities [13]. Effective contraception is required during treatment and for three years after discontinuation, and acitretin should not be used during breastfeeding [13,62,63].

Cyclosporine may be considered during pregnancy when systemic treatment is clinically necessary, particularly in severe psoriasis [13,62,63]. Although it crosses the placenta, available evidence has not demonstrated a clear increase in congenital malformations. Monitor maternal blood pressure and renal function during treatment. Decisions regarding its use during breastfeeding should follow current guidelines and product-specific recommendations.

In patients with PsA, sulfasalazine is compatible with pregnancy when clinically indicated, with appropriate folic acid supplementation. Leflunomide should be discontinued before conception and avoided during pregnancy because of the prolonged persistence of its active metabolite; an accelerated elimination procedure with cholestyramine may be required according to current recommendations. Apremilast is not recommended during pregnancy because of insufficient human safety data.

Systemic glucocorticoids are not routinely recommended for plaque psoriasis and are used only rarely in selected clinical situations. In PsA, systemic glucocorticoids are used more frequently and may be considered for short-term control of clinically significant inflammatory activity. During pregnancy, avoid prolonged or high-dose treatment whenever possible, and use the lowest effective dose for the shortest necessary duration [63].

Biologic Agents

Biologic agents are increasingly important in managing moderate-to-severe psoriasis and PsA in women of childbearing age. Pregnancy safety evidence comes primarily from registries and observational studies, with the most extensive experience for TNF inhibitors (TNFi) [13,63].

Placental transfer differs between biologic agents and increases with gestational age for IgG-containing antibodies because of Fc receptor-mediated transport. Adalimumab and infliximab undergo substantial active placental transfer, particularly during the second and third trimesters, whereas etanercept transfer is lower. Certolizumab pegol lacks an Fc region and therefore shows minimal placental transfer. Its principal advantage is low fetal exposure, making it a useful option when continuous biologic treatment is required throughout pregnancy; however, it should not be regarded simply as the “safest” biologic agent [13].

Available evidence has not demonstrated an increased risk of major congenital malformations attributable to TNFi. Current European Alliance of Associations for Rheumatology (EULAR) recommendations indicate that all TNFi may be used throughout pregnancy when clinically required, following an individualized risk-benefit assessment that considers maternal disease activity and the consequences of treatment withdrawal [38,66]. TNFi are also considered compatible with breastfeeding [38].

Placental transfer differs substantially among individual TNFi and increases with advancing gestation for Fc-containing monoclonal antibodies. Infliximab and adalimumab undergo substantial active placental transfer, whereas etanercept transfer is lower. Certolizumab pegol lacks an Fc region and demonstrates no to minimal placental transfer; therefore, its principal advantage is the very low level of fetal exposure when continuous TNFi is required throughout pregnancy [67,68]. It should therefore not simply be described as the “safest” biologic agent [67].

Recommendations regarding live vaccines in infants exposed to biologic therapy in utero should be based on the individual biologic and the timing of fetal exposure. According to BSR guidance, infliximab may be discontinued at 20 weeks, adalimumab and golimumab at 28 weeks, and etanercept at 32 weeks in women at low risk of disease flare, allowing a normal vaccination schedule in full-term infants. If these agents are continued throughout pregnancy, avoid live vaccines until six months of age. In contrast, certolizumab pegol has no to minimal placental transfer and does not require modification of the infant vaccination schedule [67].

Pregnancy safety data for non-TNFi biologics remain less extensive than for TNFi. For IL-17 inhibitors, including secukinumab and ixekizumab, available evidence has not demonstrated a specific teratogenic signal, although the evidence base remains smaller than that for TNFi [66,67]. Similarly, pregnancy data for IL-12/23 and IL-23 inhibitors, including ustekinumab, guselkumab, tildrakizumab, and risankizumab, remain comparatively limited. Recent EULAR recommendations state that non-TNFi biologic DMARDs may be used during pregnancy when clinically required after an individualized risk-benefit assessment [38]. This approach is particularly relevant when discontinuation of effective therapy is likely to result in clinically significant disease activity.

Available pharmacovigilance data on biologics used for psoriasis, including IL-17 and IL-23 inhibitors, have not identified a clear overall pregnancy safety signal; however, these findings should be interpreted cautiously because pharmacovigilance data cannot establish drug safety or exclude uncommon adverse outcomes [69]. Therefore, TNFi remain supported by a substantially larger pregnancy safety evidence base than newer biologic classes [66].

Transfer of monoclonal antibodies into breast milk is generally low, and systemic absorption by the breastfed infant is expected to be limited. The updated EULAR recommendations consider all biologic DMARDs compatible with breastfeeding, including TNFi and non-TNFi biologics [38]. Nevertheless, direct lactation data vary considerably among agents, particularly for newer IL-17 and IL-23 inhibitors, and treatment decisions should remain individualized [38,66].

Treatment during lactation

The treatment of psoriasis and PsA during breastfeeding requires special caution; however, compared to pregnancy, therapeutic options are much broader [11]. Table 1 also summarizes current recommendations on systemic therapies during lactation. It is crucial to assess the transfer of drugs into breast milk and the potential impact of therapy on the newborn and infant. The extent of drug transfer into breast milk depends primarily on the drug’s molecular weight, lipophilicity, degree of plasma protein binding, and oral bioavailability in the infant [11]. Drugs with high molecular weight and low oral bioavailability are generally considered safer during lactation.

Topical therapy remains the mainstay of psoriasis treatment during breastfeeding [11]. Emollients and moisturizers are completely safe and can be used without restriction [11]. Topical glucocorticosteroids are considered relatively safe, particularly low- to moderate-potency formulations [11]. However, it is recommended to avoid applying medications directly to the nipples before feeding to limit accidental ingestion of the product by the infant [11]. Similar recommendations apply to topical calcineurin inhibitors, such as tacrolimus and pimecrolimus, which have minimal systemic absorption [11]. NB-UVB phototherapy remains one of the safest methods for treating moderate-to-severe psoriasis during lactation [11]. This therapy does not affect breast milk composition.

In the case of systemic treatment, it is necessary to distinguish between drugs that are contraindicated, conditionally acceptable, and considered relatively safe during breastfeeding [11]. Methotrexate and retinoids, such as acitretin, are contraindicated during lactation due to their potential toxic effects and the possibility of drug accumulation in the infant’s body [22]. For this reason, breastfeeding while using these therapies is not recommended.

Cyclosporine is also not routinely recommended during breastfeeding, although it may be considered in selected cases under close clinical supervision [11]. The drug passes into breast milk, and available data on safety in infants remain limited [11]. If cyclosporine use is necessary, recommend an individualized assessment of benefits and risks, along with monitoring of the infant’s condition.

Systemic glucocorticosteroids may be used during lactation, particularly for short-term treatment of disease flares. Their transfer into breast milk is minimal, which limits the risk of adverse effects in the infant. However, the lowest effective dose should be used for the shortest necessary period [11]. At higher doses, some authors recommend waiting several hours between taking the medication and breastfeeding.

Biologic agents, particularly TNF-α inhibitors such as adalimumab, etanercept, and infliximab, are particularly important in systemic treatment during lactation [11]. Because of their high molecular weight, they pass into breast milk only in minimal amounts and are further degraded in the infant’s gastrointestinal tract, limiting their bioavailability. TNF-α inhibitors are currently considered among the safest biologic therapies during breastfeeding [11].

Certolizumab pegol has the best-documented safety profile, showing negligible transfer into breast milk [11]. The CRADLE study demonstrated minimal or undetectable concentrations of certolizumab pegol in the milk of breastfeeding women, confirming very low infant exposure to the drug [70]. Certolizumab is currently considered the preferred therapeutic option for breastfeeding women requiring biologic therapy, particularly in cases of active PsA [11,70].

Safety data on IL-17, IL-23, and IL-12/23 inhibitors during lactation remain limited [11]. No clear adverse effects have been demonstrated in infants breastfed by mothers using these therapies; however, the number of available observations is small [11]. For this reason, decisions should be made on an individual basis, following a thorough analysis of the benefits and potential risks.

Current recommendations also emphasize maintaining adequate control of disease activity during the postpartum period [63]. Uncontrolled psoriasis or active PsA can negatively affect the mother’s general condition, increase the risk of severe disease flares, and hinder care for the newborn [63]. For this reason, appropriately selected anti-inflammatory treatment may be more beneficial than completely discontinuing therapy and losing control over the inflammatory process [63].

In clinical practice, decisions about continuing treatment during lactation should consider disease activity, the risk of relapse after discontinuing therapy, available safety data, and the patient’s preferences [11,25,62-64]. In most cases, continued breastfeeding is possible with appropriately selected anti-inflammatory therapy. Treatment decisions should be made individually, in collaboration with a dermatologist, rheumatologist, and pediatrician.

Pregnancy planning, preconception counseling in women with psoriasis and PsA and interdisciplinary care

**Figure 1:**
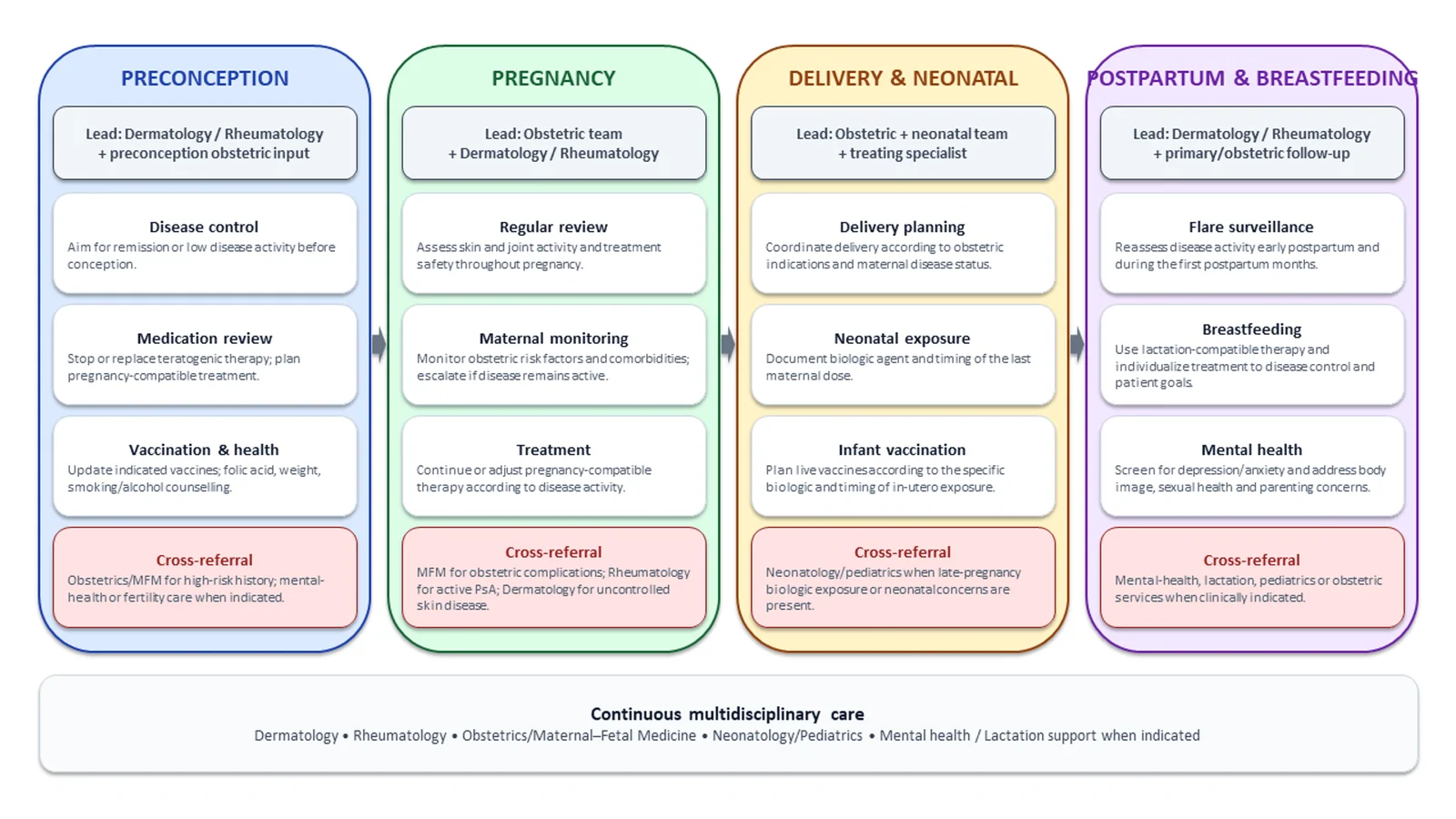
Proposed framework for the management of women with psoriasis and PsA throughout the reproductive journey The framework summarises key considerations during the preconception period, pregnancy, the postpartum period, and breastfeeding within a multidisciplinary care model [1,3,4,10,11,13,22,25,37,38]. PsA: psoriatic arthritis Image Credit: Authors using PowerPoint (Microsoft Corp., Redmond, WA, USA)

Psoriasis and PsA during pregnancy represent a significant clinical challenge due to the variable course of disease and the need to balance effective maternal treatment with fetal safety. Current evidence indicates that uncontrolled disease activity, particularly in moderate-to-severe psoriasis and PsA, is associated with an increased risk of adverse pregnancy outcomes. Adequate disease control before conception and throughout pregnancy, based on an individualized risk-benefit assessment, is essential to optimize maternal and fetal outcomes. Preconception counseling is therefore an essential part of care for women with psoriasis and PsA. Figure 1 presents an integrated framework for managing women with psoriasis and PsA across preconception, pregnancy, the postpartum period, and lactation. Current guidelines recommend planning a pregnancy when the disease is in remission or at a low activity level and while receiving treatment that is compatible with pregnancy [37,70].

Before conception, review disease activity, comorbidities, current treatment, vaccination status, and reproductive plans. Methotrexate is contraindicated during pregnancy and should be discontinued before conception according to current guidelines and product-specific recommendations. Acitretin is also contraindicated because of its prolonged teratogenic potential, requiring effective contraception during treatment and for three years after discontinuation [37,70].

Contraceptive counseling should be an integral part of preconception care for women receiving potentially teratogenic therapies. Effective contraception is strongly recommended during treatment with methotrexate, acitretin, apremilast, deucravacitinib, tofacitinib, and upadacitinib, as these agents are either contraindicated during pregnancy or there are insufficient safety data. Current recommendations favor long-acting reversible contraceptive methods, such as intrauterine devices and subdermal progestin implants, as they offer the greatest contraceptive effectiveness, with failure rates below 1% per year, and do not interfere with psoriasis or PsA treatment. Combined hormonal contraceptives and progestin-only contraceptives may also be used when not otherwise contraindicated [37,67,70].

Maintaining disease control is generally preferred to discontinuing an effective therapy. Certolizumab pegol is the preferred biologic agent because it has minimal placental transfer and can be continued throughout pregnancy. Adalimumab, infliximab, etanercept, and golimumab may also be continued as necessary to maintain disease control [37,38].

Assess vaccination status before conception. Women planning a pregnancy should have documented immunity against measles, mumps, and rubella (MMR) and varicella. Women who are not immune should receive the MMR vaccine and complete the two-dose varicella vaccine schedule at least four weeks before conception, since both vaccines contain live attenuated viruses and are contraindicated during pregnancy. Annual inactivated influenza vaccination and vaccination against SARS-CoV-2 are recommended before and during pregnancy, as they reduce the risk of severe maternal infection, hospitalization, and adverse pregnancy outcomes. Vaccination against hepatitis B should be considered for all non-immune women, especially prior to the initiation of biologic or immunosuppressive therapy. Ideally, human papillomavirus vaccination should be completed according to national immunization schedules before conception. Current rheumatology and dermatology guidelines also recommend reviewing vaccination status before initiating biologic therapy, as live vaccines are generally contraindicated during treatment. Vaccination should be completed before immunosuppression whenever possible [37,38,71].

Lifestyle optimization, including smoking cessation, avoiding alcohol, managing weight, and controlling metabolic comorbidities, should be encouraged. Folic acid supplementation should be started at least one month before conception. The recommended daily dose is 0.4 mg for women at average risk and 4-5 mg for high-risk patients, including those with a recent history of methotrexate exposure or sulfasalazine treatment, or who are obese, diabetic, taking antiepileptic therapy, or have a history of neural tube defects [37,71].

To optimize disease control, adjust treatment, and improve maternal and fetal outcomes, a multidisciplinary approach involving dermatologists, rheumatologists, obstetricians, and psychologists is recommended. Further prospective studies and pregnancy registries are needed to better define the safety and optimal use of systemic and biologic therapies in this population.

Practical implications and directions for future research

Psoriasis and PsA in women of childbearing age represent a significant clinical problem requiring multidisciplinary care from dermatologists, rheumatologists, and obstetricians. Current data indicate that adequate control of disease activity before and during pregnancy is crucial for reducing the risk of maternal-fetal complications. Simionescu et al. emphasize that “careful pregnancy monitoring in moderate-to-severe psoriasis vulgaris is mandatory,” highlighting the need for close monitoring of pregnant women with moderate-to-severe disease [1].

The literature increasingly highlights how chronic inflammation affects pregnancy. The authors emphasize that the course of pregnancy in women with psoriasis depends on many factors, such as disease activity, cytokine burden, comorbidities, and treatment [1]. Maintaining disease remission even before planned conception is particularly important, as an active inflammatory process may increase the risk of preterm birth, gestational hypertension, preeclampsia, and low birth weight in the newborn [13].

A growing body of evidence suggests that PsA may increase obstetric risk. Available studies have shown that active PsA during pregnancy may increase the risk of developing preeclampsia [72]. The mechanism likely involves chronic immune activation and increased pro-inflammatory cytokines, such as IL-17 and IL-23, which may also contribute to the pathogenesis of hypertensive disorders of pregnancy [1].

The safety of therapies used during pregnancy remains an important area of uncertainty. The best-documented are TNF-α inhibitors, particularly certolizumab pegol, which current guidelines may allow throughout pregnancy [72]. At the same time, for drugs such as secukinumab or ustekinumab, it is recommended to consider discontinuing therapy due to the limited safety data available [72]. The safety of IL-17 and IL-23 inhibitors, as well as new targeted therapies, remains insufficiently understood, which creates difficulties in making therapeutic decisions for women planning pregnancy.

The literature also emphasizes the need to establish multicenter pregnancy registries that include patients with psoriasis and PsA treated with biologics. Kurizky et al. note that the risk to the fetus stems from both the disease activity itself and the treatment used to control it [13]. Most data currently come from observational studies, case reports, and small clinical cohorts, which limits the ability to formulate definitive treatment recommendations.

Despite advances in knowledge, significant gaps remain regarding the impact of psoriasis and PsA activity on pregnancy outcomes and the long-term safety of modern biologic therapies. Managing psoriasis during pregnancy remains challenging because prospective clinical trials rarely include pregnant women, and available recommendations largely rely on observational studies, pregnancy registries, case reports, and limited clinical evidence [62,73]. Further prospective studies and long-term follow-up of children exposed to biologic therapy in utero are necessary. This will enable more precise guidelines and improve the safety of both mothers and their children.

## Conclusions

The current body of evidence suggests that reproductive management in patients with psoriasis and PsA should prioritize effective disease control throughout the preconception period, pregnancy, and the postpartum period. Active systemic inflammation has been demonstrated to contribute to impaired reproductive health and adverse pregnancy outcomes. Conversely, unnecessary treatment withdrawal has been shown to increase the risk of disease exacerbation. Consequently, therapeutic decisions should be informed by an individualized assessment of disease activity, maternal and fetal risks, comorbidities, and the reproductive safety profile of available therapies. TNFi have the most well-established safety data among biologic agents, whereas evidence for newer targeted therapies, particularly IL-17 and IL-23 inhibitors, remains comparatively limited. It is imperative to acknowledge the significant knowledge gaps that persist in relation to fertility, paternal drug exposure, and the independent contribution of disease activity and associated comorbidities to adverse reproductive outcomes.

Further prospective studies and adequately powered pregnancy registries are required to define the reproductive safety of newer therapies and to evaluate long-term maternal and offspring outcomes. Future research should incorporate fertility, postpartum disease activity, breastfeeding, patient-reported outcomes, and conventional obstetric endpoints. Optimal management requires preconception counseling, maintenance of adequate disease control with pregnancy- and lactation-compatible therapies, and coordinated multidisciplinary care involving dermatologists, rheumatologists, obstetricians, and other specialists when indicated.
